# epiTCR-KDA: knowledge distillation model on dihedral angles for TCR-peptide prediction

**DOI:** 10.1093/bioadv/vbae190

**Published:** 2024-11-29

**Authors:** My-Diem Nguyen Pham, Chinh Tran-To Su, Thanh-Nhan Nguyen, Hoai-Nghia Nguyen, Dinh Duy An Nguyen, Hoa Giang, Dinh-Thuc Nguyen, Minh-Duy Phan, Vy Nguyen

**Affiliations:** Faculty of Information Technology, University of Science, Ho Chi Minh City, Vietnam; Vietnam National University, Ho Chi Minh City, Vietnam; Medical Genetics Institute, Ho Chi Minh City, Vietnam; Bioinformatics Institute, A*STAR, Singapore; Medical Genetics Institute, Ho Chi Minh City, Vietnam; Medical Genetics Institute, Ho Chi Minh City, Vietnam; Department of Genetics and Genomic Sciences School of Medicine, Case Western Reserve University, Cleveland, Ohio, United States; Medical Genetics Institute, Ho Chi Minh City, Vietnam; Faculty of Information Technology, University of Science, Ho Chi Minh City, Vietnam; Vietnam National University, Ho Chi Minh City, Vietnam; Medical Genetics Institute, Ho Chi Minh City, Vietnam; NexCalibur Therapeutics, DE, United States; Medical Genetics Institute, Ho Chi Minh City, Vietnam

## Abstract

**Motivation:**

The prediction of the T-cell receptor (TCR) and antigen bindings is crucial for advancements in immunotherapy. However, most current TCR-peptide interaction predictors struggle to perform well on unseen data. This limitation may stem from the conventional use of TCR and/or peptide sequences as input, which may not adequately capture their structural characteristics. Therefore, incorporating the structural information of TCRs and peptides into the prediction model is necessary to improve its generalizability.

**Results:**

We developed epiTCR-KDA (KDA stands for Knowledge Distillation model on Dihedral Angles), a new predictor of TCR-peptide binding that utilizes the dihedral angles between the residues of the peptide and the TCR as a structural descriptor. This structural information was integrated into a knowledge distillation model to enhance its generalizability. epiTCR-KDA demonstrated competitive prediction performance, with an area under the curve (AUC) of 1.00 for seen data and AUC of 0.91 for unseen data. On public datasets, epiTCR-KDA consistently outperformed other predictors, maintaining a median AUC of 0.93. Further analysis of epiTCR-KDA revealed that the cosine similarity of the dihedral angle vectors between the unseen testing data and training data is crucial for its stable performance. In conclusion, our epiTCR-KDA model represents a significant step forward in developing a highly effective pipeline for antigen-based immunotherapy.

**Availability and implementation:**

epiTCR-KDA is available on GitHub (https://github.com/ddiem-ri-4D/epiTCR-KDA).

## 1 Introduction 

Immunotherapy has become a preferred treatment for certain types of tumors by harnessing the body’s immune system to recognize and destroy cancer cells. One approach to immunotherapy focused on immune checkpoint blockade (ICB), which employs monoclonal antibodies to block checkpoint proteins—such as PD-1, PD-L1, or CTLA-4—from binding to their ligands, thereby allowing T cells to target cancer cells (Shiravand *et al.* 2022, Yin *et al.* 2023). While ICB has demonstrated success in treating several solid tumors, patient responses can vary, likely due to differences in T cell recognition of tumor antigens (Sun *et al.* 2023). Emerging research suggests that enhancing the activity of reactive T cells targeting patient-specific tumor neoantigens could significantly improve the efficacy of checkpoint inhibitors, marking a promising new direction in cancer immunotherapy (Zhu *et al.* 2021). As a result, accurately predicting the interaction between T cell receptors (TCRs) and neoantigens presented by human leukocyte antigen molecules is essential for identifying therapeutic peptides used in immunotherapy.

Multiple attempts have been made to create prediction tools for TCR-peptide binding using diverse computational approaches. There are simple models such as Bayesian approach [TCRGP (Jokinen *et al.* 2021), TCR-Pred (Smirnov *et al.* 2023)], Random Forest [TCRex (Gielis *et al.* 2019), epiTCR (Pham *et al.* 2023)], and clustering-based models [TCRdist (Dash *et al.* 2017)]. More complex models (Moris *et al.* 2021, Sidhom *et al.* 2021) are also proposed for the classification task. Many deep learning models [NetTCR (Montemurro *et al.* 2021), DeepTCR (Sidhom *et al.* 2021), ImRex (Moris *et al.* 2021), tcrpred (Koyama *et al.* 2023)] rely on convolutional neural networks (CNN) to learn the TCR and peptide patterns in each interaction. Some other tools, particularly ERGO-I (Springer *et al.* 2020) and pMTnet (Lu *et al.* 2021), use long short-term memory to learn the sequential information of TCR and peptides, and autoencoder layers to simultaneously improve the data understanding and reduce the feature space. Also extracting sequence information, BERTrand (Myronov *et al.* 2023), a language model-based model, learns the amino acid position and composition in the TCR and peptide sequences contributing to the binding. Despite many machine learning and deep learning algorithms that have been applied to predict the interactions between TCR and peptides, predicting the TCR-peptide binding is still a challenge, especially when applying to unseen data where either the sequences of TCR or peptide or both are not presented in the training dataset.

Most TCR-peptide binding predictors struggle to generalize the interaction of TCR and peptide (Lu *et al.* 2021, Sidhom *et al.* 2021, Grazioli *et al.* 2023). The first reason is the datasets used to train and test predictive models are limited in size or diversity, particularly when it comes to the number of peptides. It was demonstrated with NetTCR that there was a positive correlation between the model performance and the size of the training dataset (Montemurro *et al.* 2021). The available data may not represent the full spectrum of peptide variability or skewed towards certain peptide sequence patterns (Grazioli *et al.* 2022). Furthermore, some studies (Montemurro *et al.* 2021, Springer *et al.* 2021) suffered from overoptimistic classification performance when using random data split for training and testing, inadvertently resulting in data leakage due to the presence of the same peptide sequences in both training and testing data even though the TCR-peptide pairs are not overlapping (Grazioli *et al.* 2022). Therefore, careful attention is needed in dataset construction and validation, with a special focus on using unseen peptides for testing, to prevent data leakage and ensure the development of robust predictive models. The second reason is the insufficient information for the models to learn from the input pair of the amino acid sequences of the TCR CDR3β region and the peptide, of which the linear sequences do not represent the spatial information of the TCR-peptide interactions. In fact, two different TCR-peptide sequence pairs can share similar spatial information and, therefore, can interact in the same manners (Moris *et al.* 2021). This lack of spatial information might prevent models from generalizing the TCR-peptide interactions, leading to low performance on unseen data.

Many protein structure prediction tools, such as AlphaFold (Jumper *et al.* 2021), ESMFold (Lin *et al.* 2023), PEP-FOLD3 (Lamiable *et al.* 2016), and OmegaFold (Wu *et al.* 2022), can transform linear amino acid sequence to spatial information, which better represents the biological interaction of TCRs and peptides [TCR-Pred (Smirnov *et al.* 2023)]. However, the full 3D structure data for every single atom of each amino acid is a complex, high-dimensional set of data for model input, which can exponentially complicate the learning process. To address this, the dihedral angles, including the phi (*ϕ*) angle around the backbone N-Cα bond and the psi (*ψ*) angle around the backbone Cα-C bond, can be used to represent the 3D shape of a peptide (Knapp *et al.* 2008, Ferber *et al.* 2012). These dihedral angles can serve as effective features to capture the structural information of both the TCR CDR3β and peptide, guiding the models to learn the patterns of spatial interactions.

In this study, we present epiTCR-KDA (KDA stands for Knowledge Distillation model on Dihedral Angles), a novel approach to predict the TCR-peptide binding based on a knowledge distillation model (KD) (Hinton *et al.* 2015), which learns the spatial information from dihedral angles of both the TCR CDR3β and peptides. The epiTCR-KDA was trained on a dataset of diverse TCR and peptides, with additional known non-binding peptides (wild type) sourced from public databases. The model consistently outperformed other currently available TCR-peptide binding prediction tools. Furthermore, our epiTCR-KDA also demonstrated outstanding generalization ability on unseen data.

## 2 Methods

### 2.1 Data collection and generation of non-binding TCR-peptide pairs

**Figure 1.**
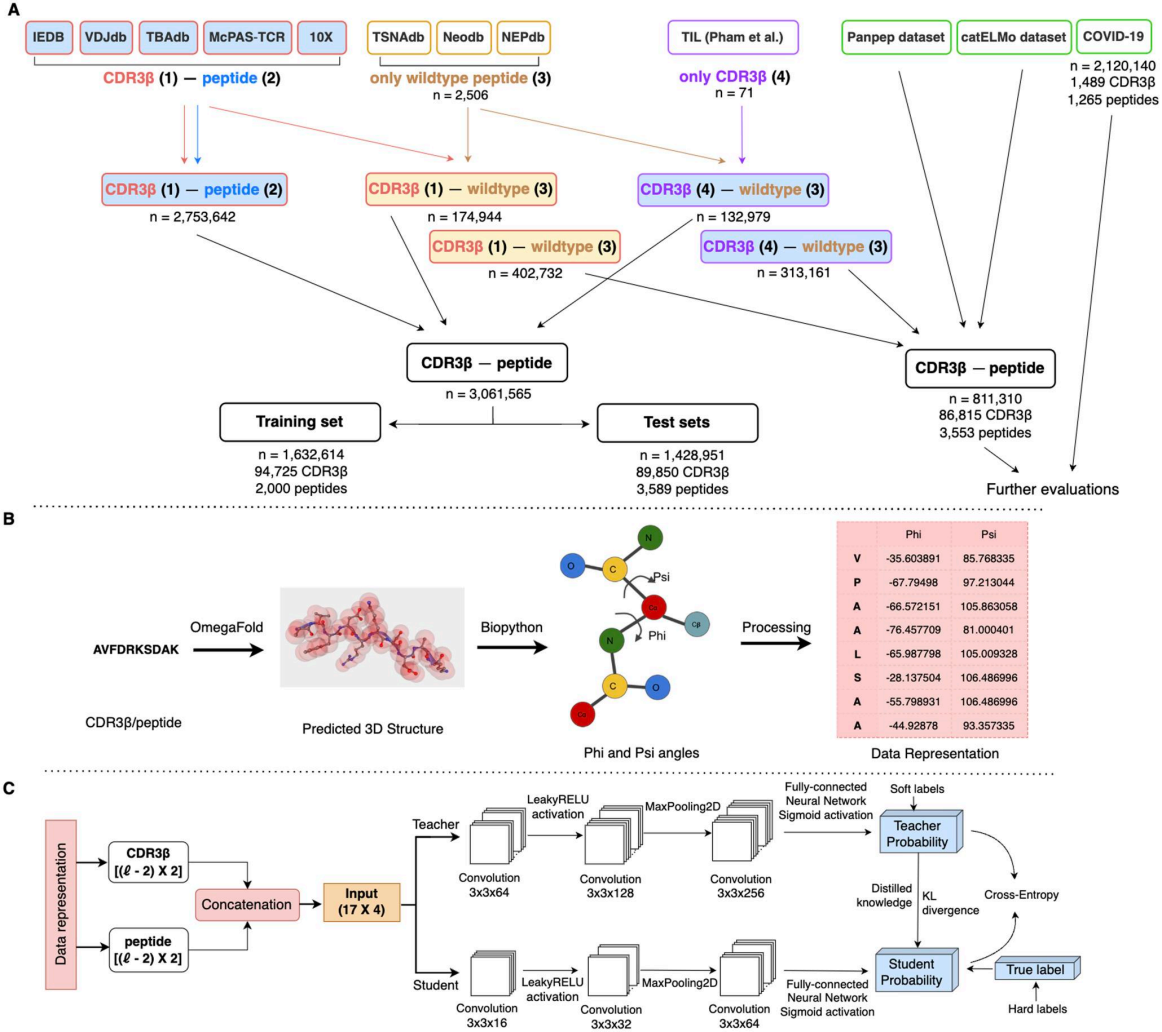
Overview of epiTCR-KDA. (A) Diagram illustrating data collection for training and evaluation of epiTCR-KDA. Five public databases [IEDB (Vita *et al.* 2019), VDJdb (Shugay *et al.* 2018), TBAdb (Zhang *et al.* 2020), McPAS-TCR (Tickotsky *et al.* 2017), and 10X (*A New Way of Exploring Immunity—Linking Highly Multiplexed Antigen Recognition to Immune Repertoire and Phenotype | Technology Networks*, 2020)] were collected for TCR-peptide pairs, with publicly collected TCR labeled as (1) and publicly collected peptides labeled as (2) (Supplementary Table S1). Three databases [TSNAdb ( Wu *et al.* 2023a), Neodb (Wu *et al.* 2023b), and NEPdb (Xia *et al.* 2021)] were gathered for self-peptides (wildtype peptides), labeled as (3). These peptides were randomly combined with TIL TCR, labeled as (4), from public TCR-peptide pairs to form non-binding pairs [i.e. (3) combined with (4)]. Additionally, non-binding pairs were also generated from TIL CDR3β sequences with public wildtype peptides [i.e. (1) combined with (3)]. The data were divided into training data (Supplementary Fig. S2), and testing data covering various data sources, seen and unseen peptides (Supplementary Table S2). (B) Data preprocessing steps starting from the conversion of CDR3β/peptide amino acid sequences to 3D structures using OmegaFold, followed by the calculation of the phi and psi angles, and processing this information as input for the model (Supplementary Fig. S1). (C) Structure of the KD model. The CDR3β and peptide representation (phi and psi angles) were concatenated, padded, and served as input for the KD model. The KD model involved a student model learning from the information provided by the teacher model (soft loss) and ground-truth labels (hard loss). The model was trained to predict the binding or non-binding of CDR3β-peptide pairs.

The CDR3β loop plays a key role in the TCR-peptide interactions (Reiser *et al.* 2002, Tsuchiya *et al.* 2018, Croce *et al.* 2024), and public data mostly contains CDR3β-peptide interactions. Previous study also highlighted CDR3β as a good representative for TCRs (Moris *et al.* 2021, Grazioli *et al.* 2023, Pham *et al.* 2023, Ji *et al.* 2024). Therefore, in this work, we continue to use the information from the CDR3β loop to improve the prediction of TCR-peptide binding. Binding and non-binding CDR3β-peptide pairs were collected from McPAS-TCR (Tickotsky *et al.* 2017), TBAdb (Zhang *et al.* 2020), VDJdb (Shugay *et al.* 2018), IEDB (Vita *et al.* 2019), and 10X (*A New Way of Exploring Immunity—Linking Highly Multiplexed Antigen Recognition to Immune Repertoire and Phenotype | Technology Networks*, 2020). The combined dataset contains 70 083 (2.5%) binding pairs and 2 689 709 non-binding pairs that were formed by 1681 unique peptides and 126 841 unique CDR3β sequences. Among 1681 unique peptides, only 7 are exclusively found in non-binding pairs, significantly lower than the number of peptides exclusively found in binding pairs (1637 unique peptides). The small number of unique peptides in non-binding pairs highlighted the data imbalance, leading to bias towards the positive “binding pairs” predictions (Pham *et al.* 2023). To address this issue, we augmented the data by constructing additional non-binding CDR3β-peptide pairs. We first extracted wildtype peptide sequences from TSNAdb (Wu *et al.* 2023a), Neodb (Wu *et al.* 2023b), and NEPdb (Xia *et al.* 2021), and then randomly combined them with CDR3β sequences from previously collected data. This resulted in additional 174 944 CDR3β-peptide pairs with 2506 unique peptides for the non-binding dataset. On the other hand, we further combined 71 CDR3β sequences from tumor-infiltrating T cells (TIL) (Pham *et al.* 2024) with the extracted wildtype peptides to make additionally 132 979 non-binding CDR3β-peptide pairs. Details can be found in Fig. 1A.

### 2.2 Input data representation

The phi (*ϕ*) and psi (*ψ*) torsion angles were used to represent the structural information of both the CDR3β and peptide sequences. The CDR3β/peptide sequences were first used to predict their 3D-structures using OmegaFold (version v1.1.0) (Wu *et al.* 2022). The phi and psi angles were then calculated using the PDBParser function implemented in the biopython package (version 1.75) (*Bio.PDB.Internal_coords Module–Biopython 1.84.Dev0 Documentation*, n.d.), resulting in (l-2, 2) matrices, with l corresponding to the length of the sequence. The first and the last amino acids in the sequence can rotate freely around the peptide backbone, therefore, their phi and psi angles were excluded. The CDR3β-representing matrix and the peptide-representing matrix was calculated separately, then zero-padded to the dimension of the longest amino acid sequences of CDR3β and peptides. The two matrices were then concatenated vertically to form a (l-2, 4) matrix, with l corresponding to the longest amino acid sequence (l=17 in this study) (Supplementary Fig. S1). The resulting phi and psi matrix was provided to the learning model as input (Fig. 1B).

### 2.3 Data organization for model training and testing

The training data consisted of 1 632 614 CDR3β-peptide pairs, including 94 725 unique CDR3β sequences, and 2000 unique peptides. The testing data comprised of 1 428 951 pairs, including 89 850 unique CDR3β sequences and 3589 unique peptides. Of the unique peptides in the testing data, 1948 (54.2%) were seen peptides (peptides paired with other CDR3β in the training data), and 1641 (45.8%) were unseen peptides (peptide sequences only found in the testing data, Supplementary Table S2). The testing data were randomly split into ten testing sets, allowing the benchmark of epiTCR-KDA against other predictors. A “7 unseen dominant peptides” dataset consisting of 447 398 CDR3β-peptide pairs derived from 7 unseen peptides (Supplementary Table S7) was also randomly split into 10 subsets and used to testing the models.

### 2.4 Model training

The model structure followed a knowledge distillation approach (Hinton *et al.* 2015), akin to a teacher-student relationship (Fig. 1C). The input CDR3β and peptide sequences were individually represented by matrices of phi and psi angles, which were then concatenated and padded by zeros into a 17 × 4 matrix (where 17 rows representing the dimension obtained from the longest sequence, and 4 columns representing the phi and psi angle pairs of the CDR3β then of the peptide, respectively). Taking this matrix as input, both the student and teacher models were built based on the CNNs framework. The teacher model was for binary classification, started from a convolutional layer of 64 filters of size 3 × 3 [with the stride of (2, 2)], followed by a LeakyReLU activation (*α*  =  0.2), and a MaxPooling2D with 2 × 2 filter and stride = 1. Two convolutional layers with 128 and 256 filters (with the same filter size and stride) were subsequently applied. The output from the last layer was flattened into a 1D vector, followed by a fully connected layer, and a single unit of sigmoid activation for binary classification. The student model replicates the teacher's prediction with reduced complexity by reusing three convolutional layers of 16, 32, and 64 filters, respectively, while other layers were kept similar to those of the teacher model. The distillation involved a Distiller object containing both models. During training, the Distiller object was compiled using Adam optimizer, with BinaryAccuracy metric for evaluation, BinaryCrossentropy loss function for the student, and KLDivergence for distillation loss evaluation. These parameters resulted from the model tuning process.

## 3 Results

### 3.1 Overview of epiTCR-KDA

To construct a predictive model for TCR-peptide binding, we tackled the problem from three main angles: data collection, data encoding, and model structure. Different training datasets can significantly impact the model's performance, so we focused on obtaining diverse CDR3β sequences and peptides with known binding status from multiple public sources (Fig. 1A). Additionally, we generated non-binding pairs to increase the proportion of non-binding data, based on the assumption that TCRs do not bind to or activate against human wildtype (self) peptides (Fig. 1A). For the training data, we generated a series of sets with an increasing number of peptides and corresponding CDR3β-peptide pairs and found that a set with 2000 unique peptide sequences exhibited the best training performance (Supplementary Fig. S2). The final training data consisted of 1 632 614 CDR3β-peptide pairs, comprising 94 725 unique CDR3β sequences and 2000 unique peptides (Fig. 1A). We hypothesized that traditional amino acid sequence-based encoding methods, such as one-hot encoding or BLOSUM62, might not provide sufficient insights into the 3D structures of the two binding partners. Therefore, we used dihedral angles as input data to better capture the structural information of the CDR3β and peptides (Fig. 1B, Supplementary Fig. S1). Then, a knowledge distillation (KD) model was used to learn from the dihedral angle matrix input (Fig. 1C). The KD process involved a more complex “teacher” model, extracting deep-level details from the TCR and peptide structures, and then transferring that knowledge to a smaller, simpler “student” model. This allowed the student model to reduce any overfitting that might have occurred in the teacher model. Ultimately, the KD model was used to enhance the generalization capacity of the epiTCR-KDA approach.

### 3.2 epiTCR-KDA outperformed existing tools in predicting the binding of unseen peptides

**Figure 2.**
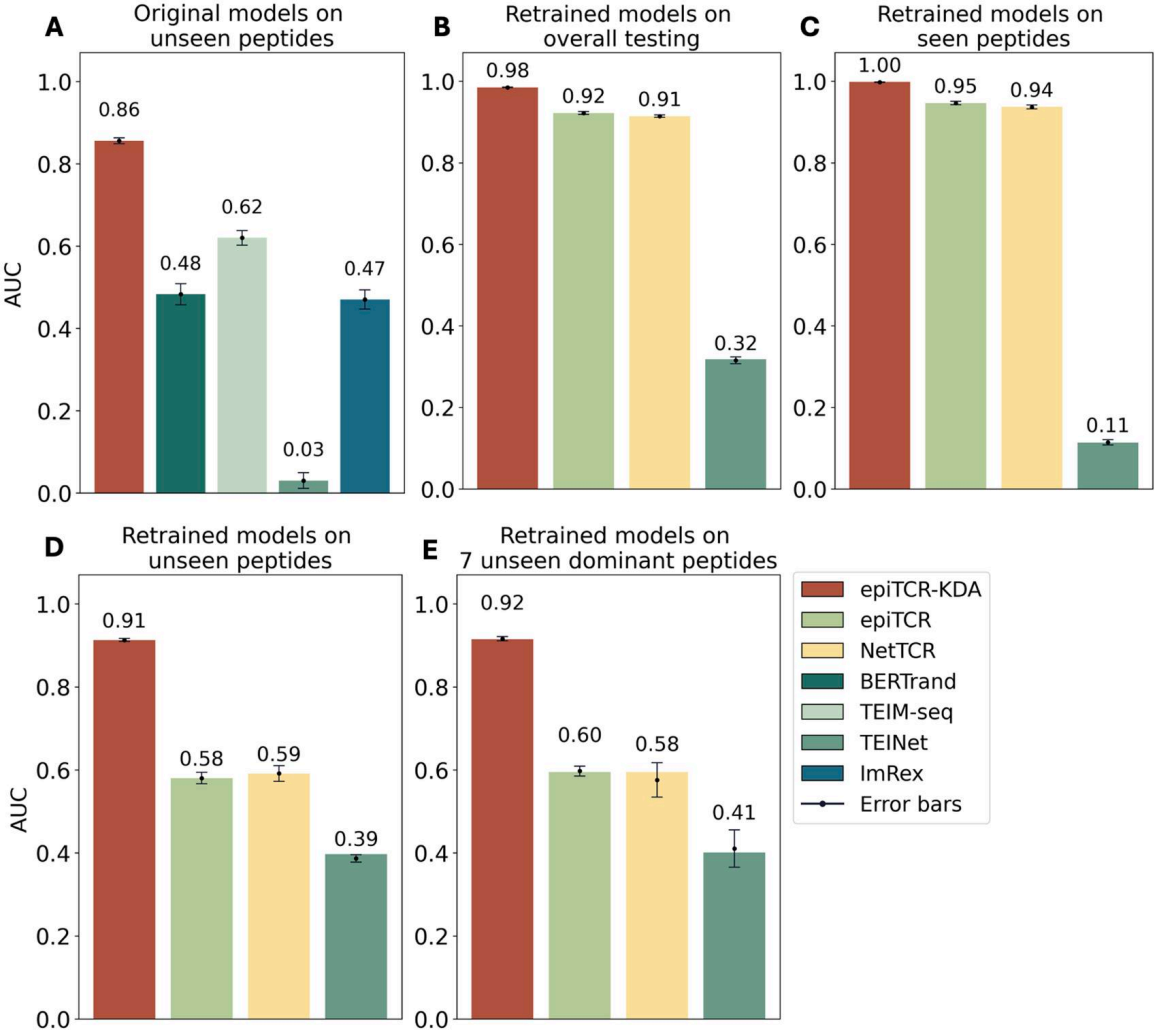
The performance of epiTCR-KDA, epiTCR, NetTCR, BERTrand, TEIM-Seq, TEINet, and ImRex across different benchmark settings: (A) original models tested on 10 datasets containing peptides unseen from training of all those models, (B) retrained models on 10 overall testing sets including both seen and unseen data, (C) retrained models on data derived from seen peptides, (D) retrained models on data derived from seen peptides, and (E) retrained models on data derived from 7 dominant unseen peptides (Supplementary Table S7). The performance was measured by AUC. Each bar indicates the mean performance from ten testing sets and the error bar indicates the standard deviation. The original models of epiTCR and NetTCR were also benchmarked on interactions of unseen peptides; however, epiTCR produced only positive predictions, while NetTCR gave only negative predictions for all interactions. Consequently, AUC was not calculated for epiTCR and NetTCR in this testing scenario (Supplementary Table S5).

To compare the performance of epiTCR-KDA with currently available tools, we chose a set of benchmarked predictors covering a wide range of data representation and learning approaches, including BERTrand (Myronov *et al.* 2023), TEIM-Seq (Peng *et al.* 2023), TEINet (Jiang *et al.* 2023), ImRex (Moris *et al.* 2021), epiTCR (Pham *et al.* 2023), and NetTCR (Montemurro *et al.* 2021), all of which use the CDR3β and peptide sequences as input. First, we used the original published model of each tool to benchmark against epiTCR-KDA. Because the training data for each model was different, we designed 10 testing sets that were unseen by all models for fair comparison (see Supplementary Methods). In this benchmark, our epiTCR-KDA model achieved an average area under the curve (AUC) of 0.86, far exceeding the performance of next model, TEIM-seq, at 0.62 (Fig. 2A, Supplementary Tables S4 and S5).

Second, to provide a better benchmark, we decided to retrain the models using the same training data as used for epiTCR-KDA before comparing their performance. Only three models, epiTCR, NetTCR and TEINet, were retrained thanks to the availability of training codes from the authors (Supplementary Methods). These three models were benchmarked against epiTCR-KDA using 10 non-overlapping testing sets, randomly sampled from the testing data consisting of 1 428 951 CDR3β-peptide pairs. Each testing set contained 60% seen data (pairs of CDR3β-peptide in which the peptide sequences were also found in the training set) and 40% unseen data (pairs of CDR3β-peptide in which peptide sequences were only found in the testing data). A significant drop in performance from seen to unseen data indicates the low generability of a model.

Overall, epiTCR-KDA performed the best, achieving an average AUC of 0.98 (Fig. 2B, Supplementary Figs. S3E and S4E). The second and the third best-performing tools were epiTCR and NetTCR, with average AUC values of 0.92 and 0.91, respectively (Fig. 2B, Supplementary Figs. S3E and S4E). When evaluating their performance on seen data, epiTCR-KDA, epiTCR, and NetTCR showed comparable results with AUC values of 1.00, 0.95, and 0.94, respectively (Fig. 2C, Supplementary Figs. S3F and S4F). However, on unseen data, epiTCR-KDA clearly outperformed the others, achieving an average AUC of 0.91, compared to 0.58 and 0.59 from epiTCR and NetTCR, respectively (Fig. 2D, Supplementary Figs. S3G and S4G). Our epiTCR-KDA showed a modest drop in AUC from seen data to unseen data (from 1.00 to 0.91), while the other tools exhibited significant drops (0.95 to 0.58 in epiTCR and 0.94 to 0.59 in NetTCR), suggesting that epiTCR-KDA generalizes well.

To further challenge the models, we included a special testing set of 447 398 CDR3β-peptide pairs derived from 7 unseen peptides, which hereafter referred to as the “7 unseen dominant peptides” (Supplementary Table S7). These dominant peptides were known to significantly reduce the overall performance of prediction models (Montemurro *et al.* 2021, Pham *et al.* 2023), and here we reported the performance of each tool on the CDR3β-peptide pairs derived from these peptides. For all tools tested, the performances on the dominant peptides were slightly lower than those on unseen data (Fig. 2E, Supplementary Figs. S3H and S4H), confirming that data derived from the 7 unseen dominant peptides are more challenging to predict. Despite that, our epiTCR-KDA still maintained a good performance with AUC of 0.92.

To comprehensively evaluate the generalization capabilities of epiTCR-KDA, we categorized our dataset into different groups based on peptide sources, including virus, human, and other pathogen origins (Supplementary Table S6). We then assessed the performance of epiTCR-KDA in predicting CDR3β–peptide binding on both seen and unseen data. Notably, the AUC values for epiTCR-KDA predictions remained consistently robust across different peptide sources (Supplementary Fig. S5).

### 3.3 Dihedrals played a pivotal role in maintaining consistently good performance of our epiTCR-KDA

**Figure 3.**
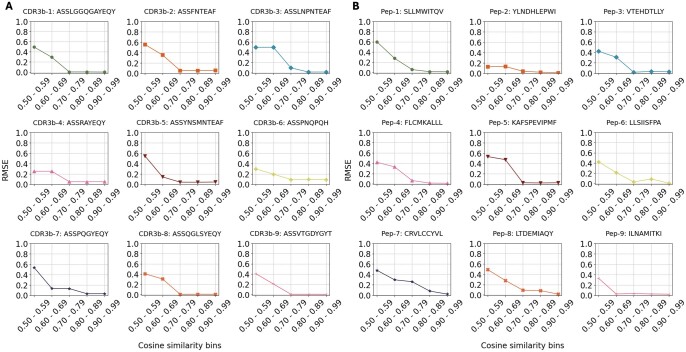
The influence of CDR3β and peptide structural information in training data on predictions by epiTCR-KDA. (A) Nine CDR3β and (B) nine peptides were chosen to represent nine clusters within the testing sets, and the predicted labels of their represented clusters were compared with the labels in training bins at different levels of dihedral angle-based cosine similarity using RMSE. The lower the RMSE, the more similar between prediction labels and training labels.

We aimed to understand the factors contributing to the consistent performance of epiTCR-KDA on both seen and unseen data. To achieve this, we evaluated the influence of training data on prediction outcomes, specifically focusing on the similarity between the TCRs and peptides in the training data versus those being predicted. We grouped each testing set into nine clusters based on their CDR3β dihedral angles. Nine representatives were used to represent the diversity of CDR3β sequences across the 10 testing sets. For each CDR3β representative, we split the training data into bins containing the CDR3β-peptide pairs, of which the respective CDR3β sequences maintained similar (i.e. in same range of cosine similarity of the phi-psi vectors) to the representative CDR3β (see Supplementary Methods). Next, we calculated the root mean squared error (RMSE) to measure the discrepancy between the labels (binding/non-binding) of the testing cluster and those of the corresponding training bin. We observed a reduction in RSME as the similarity increased across all nine tested CDR3β sequences. It was shown that the binding/non-binding CDR3β-peptide pairs predicted by the epiTCR-KDA in each testing cluster were more associated with those of the training bins exhibiting higher cosine similarity (Fig. 3A). We performed similar measurements for the peptides and observed similar patterns (Fig. 3B). Overall, these findings suggested that dihedral angles of both CDR3β and peptide could be the key features that determined the outstanding performances of the epiTCR-KDA.

### 3.4 Robust performance of the epiTCR-KDA across different testing scenarios

**Figure 4.**
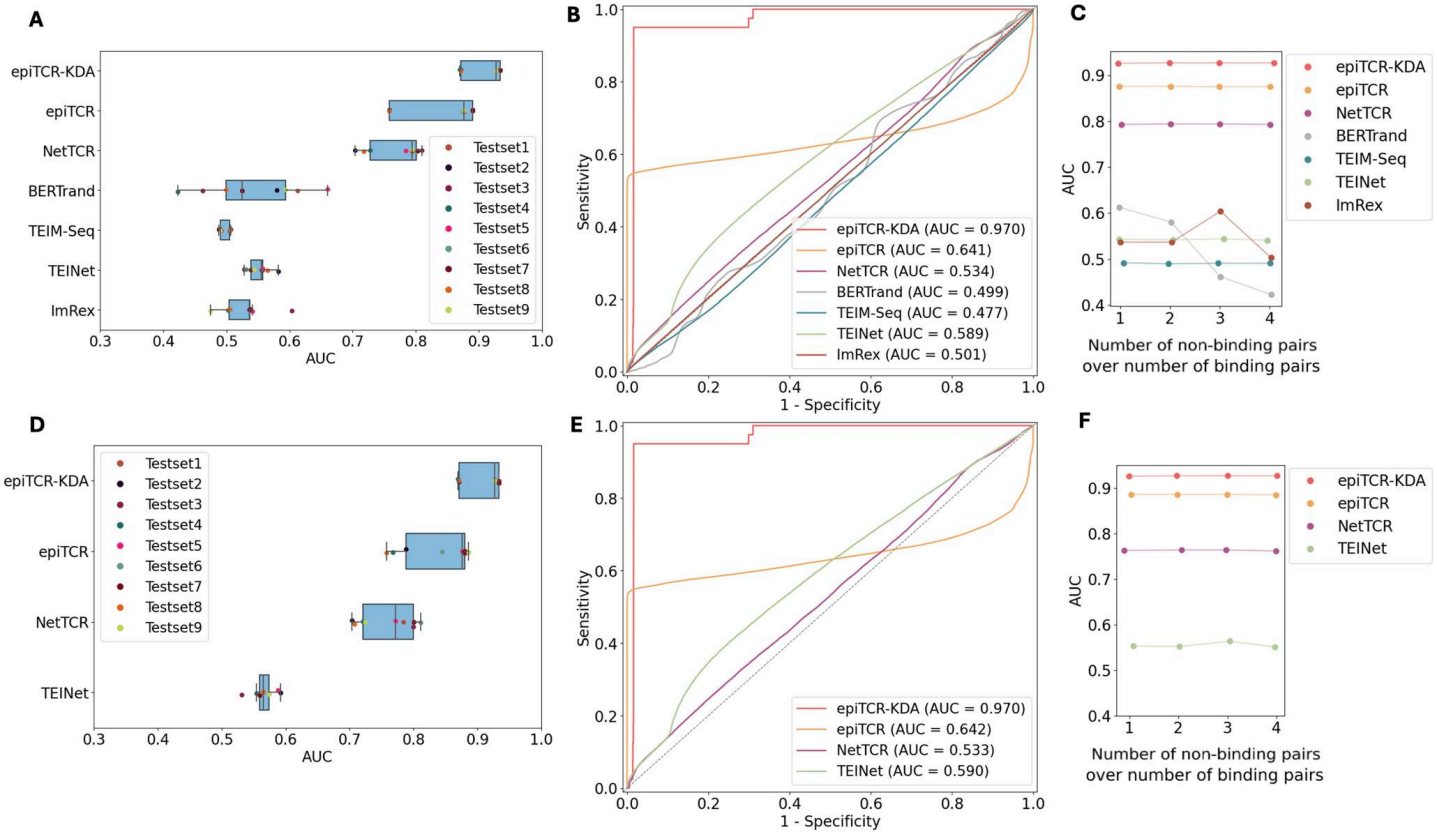
The performance different models on diverse testing scenarios. (A) epiTCR-KDA and original models on nine combined datasets, (B) epiTCR-KDA and original models on the COVID-19 dataset, and (C) epiTCR-KDA and original models on four datasets with an increasing number of non-binding pairs, (D) epiTCR-KDA and retrained models on nine combined datasets, (E) epiTCR-KDA and retrained models on the COVID-19 dataset, and (F) epiTCR-KDA and retrained models on four datasets with an increasing number of non-binding pairs.

A challenge in the TCR-peptide binding prediction is the generalizability of the prediction models, which might be varied with respect to different testing sets. We demonstrated the potential generalizability and robustness of our epiTCR-KDA by testing its performance across various datasets from multiple sources. The testing data were specifically designed to encompass diverse sources and varying ratios of non-binding to binding CDR3β-peptide pairs. The binding pairs were sourced from two studies: Panpep (Gao *et al.* 2023), with 10 397 CDR3β-peptide pairs, and catELMo (Zhang *et al.* 2023) with 85 020 CDR3β-peptide pairs. The non-binding pairs were generated as previously described (Fig. 1A) by combining public CDR3β sequences (*A New Way of Exploring Immunity—Linking Highly Multiplexed Antigen Recognition to Immune Repertoire and Phenotype | Technology Networks*, 2020; Tickotsky *et al.* 2017, Shugay *et al.* 2018, Vita *et al.* 2019, Zhang *et al.* 2020) with wildtype peptides (Xia *et al.* 2021, Wu *et al.* 2023a, 2023b). The resulting non-binding sets (*n* = 402 732 and *n* = 313 161, Fig. 1A) contained no CDR3β-peptide pairs that were present in those used in the earlier benchmark (Fig. 2). These binding and non-binding CDR3β-peptide pairs were then combined to form nine new testing datasets (Supplementary Table S8). The performance (AUC) of epiTCR-KDA and six other predictors is shown in Fig. 4A and D, with epiTCR-KDA exhibiting the median AUC of 0.93 (ranging from 0.87 to 0.93), followed by original and retrained epiTCR both achieving the median AUC of 0.88 (AUC ranging from 0.76 to 0.86 by original models, and ranging from 0.76 to 0.89 by retrained models), and original and retrained NetTCR reaching the median AUC of 0.77 and 0.79, respectively (AUC ranging from 0.7 to 0.81 by both models). It was observed that across the nine testing sets, different performances of the epiTCR-KDA were most likely attributed to the different ratios of unseen-to-seen data.

A COVID dataset (Koyama *et al.* 2023), consisting of 2 120 140 CDR3β-peptide pairs (including 2 120 100 non-binding pairs, and 40 binding pairs), was also used in our subsequent benchmark (Fig. 4B and E). This dataset includes SARS-CoV-2 peptide sequences that were not used in the training data by the epiTCR-KDA. The ratios of seen versus unseen were found in the peptides 1:125.5 and in the CDR3β sequences 1:37.7 (Supplementary Table S8). Despite the more predominant unseen data in this COVID dataset, the epiTCR-KDA performed consistently well (AUC = 0.97) in Fig. 4B and E. The next two best-performing models epiTCR and NetTCR experienced a significant drop in performance (AUC to 0.641 and 0.534 by original models, and AUC to 0.639 and 0.534 by retrained models, respectively). This result demonstrated the epiTCR-KDA generalizability on the unseen COVID data.

Subsequently, we assessed the performance of epiTCR-KDA and the other predictors using different ratios of the non-binding pairs versus the binding pairs (Fig. 4C and F, Supplementary Table S9). Generally, all the predictors (except for ImRex) performed best when this ratio was 1:1 (Fig. 4C and F). Interestingly, the top three predictors, epiTCR-KDA, epiTCR and NetTCR, consistently performed well even given the increasing ratios of the non-binding versus binding. It suggests that the epiTCR-KDA maintains its robustness.

## 4 Discussion

The potential of using neoantigens as personalized, cancer-specific markers for various therapeutic and preventative anti-cancer strategies has not been fully realized. This is partly due to the difficulties in identifying neoantigens individually for each patient. Numerous computational methods have been developed, employing a wide range of advanced deep learning models to predict TCR-peptide binding [NetTCR (Montemurro *et al.* 2021), TEIM-Seq (Peng *et al.* 2023), TEINet (Jiang *et al.* 2023), (Myronov *et al.* 2023), and ImRex (Moris *et al.* 2021)]. However, these methods typically rely on amino acid sequences as input or attempt to convert those sequences using canonical encoding techniques, such as BLOSUM (Montemurro *et al.* 2021, Pham *et al.* 2023), one-hot (Jiang *et al.* 2023), and physicochemical properties (Yang *et al.* 2023). In this study, we proposed the dihedral angles, also known as Ramachandran angles (Ramachandran *et al.* 1963), as input features to predict the TCR-peptide binding (Fig. 1). This approach is efficient and captures the three-dimensional structure of both the TCRs and peptides. Although the concept of dihedral angles is well established, to the best of our knowledge its application to predict the TCR-peptide binding pairs has not been reported previously. By providing the angular orientations of consecutive peptide bonds, we hypothesize that our model, epiTCR-KDA, could effectively learn spatial information from CDR3β and peptide, which is crucial for differentiating non-binding from binding CDR3β-peptide pairs. In fact, our epiTCR-KDA model performed consistently well across all testing scenarios (Figs. 2 and 4). This was particularly evident in cases where the number of unseen peptides far exceeded the seen peptides, as demonstrated in the COVID dataset (Fig. 4B). Our epiTCR-KDA also exhibited high generalizability.

Knowledge distillation has proven effective in various domains, such as natural language processing (Hahn and Choi 2019), computer vision (Chawla *et al.* 2021), and speech recognition (Yoon *et al.* 2021). Its versatility stems from its capacity to distill the rich knowledge captured by a complex model into a more compact representation, which is suitable for deployment in environments with limited resources. In the prediction of CDR3β-peptide binding, where accurate modelling of complex molecular interactions is essential, knowledge distillation offers a pathway to enhance the performance of simpler predictive models. By incorporating knowledge distillation with dihedral angles, our model learns from both CDR3β and peptide representations, enabling it to capture a broader range of structural features that influence binding interactions, e.g. our epiTCR-KDA exhibited substantial association of both the CDR3β and peptide similarity between training and testing data (Fig. 3). In contrast, our previous model, epiTCR, demonstrated that only 50% of the examined peptides had predicted labels similar to those of their corresponding groups of similar peptides in the training set. This finding affirms why epiTCR is less effective than epiTCR-KDA in predicting outcomes on unseen data (Fig. 2). Although we have not been able to determine whether this generalization capability is attributable to the dihedral angles input, the KD model, or a combination of both, our findings demonstrate that the epiTCR-KDA represents a promising and novel approach in the area of TCR-peptide binding prediction that has not been previously reported.

Nonetheless, several limitations remain in our current study. First, our data representation is dependent on the reliability of the OmegaFold tool (Wu *et al.* 2022) to predict the 3D structures of CDR3β and peptides. We however have not confirmed these resulting 3D models using some other tools such as AlphaFold (Jumper *et al.* 2021) and ESMFold (Lin *et al.* 2023) due to a few scenarios: (i) the short length of the CDR3β and peptide sequences do not satisfy the constraints by AlphaFold (≥16 residues), (ii) ESMFold consistently fails for certain of our sequences. Neither did we apply RosettaFold (Baek *et al.* 2021) due to our time and resource constraints. Second, our model search may not be exhaustive, hence the knowledge distillation model may not be the best model to fully capture the intricacies. Third, while our work has clearly demonstrated that incorporating 3D structure information in data representation can improve the generalizability of our model, there still remain other structural characteristics to be explored to devise more robust and versatile prediction models for diverse CDR3β-peptide complexes. Lastly, although the runtime of the epiTCR-KDA model is comparable with other tools in its class (67 seconds for 1 million input pairs), the complete process of epiTCR-KDA including transforming amino acid sequences to 3D structure requires much longer time (255 601 seconds for 1 million input pairs). OmegaFold takes approximately three seconds per amino acid sequence and prediction on a large dataset leads to notably longer runtime (Supplementary Fig. S6). Future work is needed to build a model with enhanced interpretability and fast runtime to further advance our understanding of immune system dynamics and facilitate the development of novel therapeutic strategies.

## 5 Conclusion

We presented epiTCR-KDA, a knowledge distillation model that uses dihedral angles for prediction of TCR-peptide binding. By capturing the structural information of both the partners of the TCR-peptide complexes, epiTCR-KDA elicits its generalizability and robustness across diverse datasets. Given its generalizability, the epiTCR-KDA might pave the ways for future development in the areas of immunotherapy that faces low success rate of identifying multiple personalized neoantigens capable of activating T cells.

## Supplementary Material

vbae190_Supplementary_Data
