# Supplementary material for: epiTCR-KDA: knowledge distillation model on dihedral angles for TCR-peptide prediction
Source: Bioinform Adv. 2024 Nov 29;4(1):vbae190. doi: 10.1093/bioadv/vbae190 (PMC11646569; doi:10.1093/bioadv/vbae190)
Supplement: vbae190_Supplementary_Data [file vbae190_supplementary_data.docx]

epiTCR-KDA: Knowledge Distillation model on Dihedral Angles for TCR-peptide prediction - Supplementary information

My-Diem Nguyen Pham^1,2,3^, Chinh Tran-To Su^4^, Thanh-Nhan Nguyen^3^, Hoai-Nghia Nguyen^3^, Dinh Duy An Nguyen^5^, Hoa Giang^3^, Dinh-Thuc Nguyen^1,2^, Minh-Duy Phan^3,6^, Vy Nguyen^3^

^1^ Faculty of Information Technology, University of Science, Ho Chi Minh City, Vietnam

^2^ Vietnam National University, Ho Chi Minh City, Vietnam

^3^ Medical Genetics Institute, Ho Chi Minh City, Vietnam

^4^ Bioinformatics Institute, A*STAR, Singapore

^5^ Department of Genetics and Genomic Sciences School of Medicine, Case Western Reserve University

^6^ NexCalibur Therapeutics, Delaware, US

Corresponding author:

Minh-Duy Phan

pmduy@yahoo.com

Vy Nguyen

nttv.2002@gmail.com

# Supplementary Methods

## Data collection, data preprocessing, and data generation

We presented a comprehensive analysis of CDR3β-peptide interactions by leveraging diverse data sources to gain a broad understanding of the learned model for CDR3β-peptide interactions.

First, we collected binding and non-binding CDR3β-peptide pairs from McPAS-TCR[1], TBAdb[2], VDJdb[3], IEDB[4], and 10X[5]. Each dataset underwent individual preprocessing. For CDR3β, we used amino acid sequences, and where applicable (in TBAdb, VDJdb, McPAS-TCR, and 10X datasets), we removed the “C” starting and “FW” ending characters in the CDR3β chain, similar to NetTCR[6]. In IEDB, we used curated CDR3β sequences. We also eliminated sequences containing unknown amino acids (encoded by X, O, special characters, and lowercase letters). Additionally, we removed empty peptides and unknown amino acids. We filtered all sequences based on their length, with CDR3β lengths ranging from 8 to 19 amino acids and peptide lengths from 8 to 11 amino acids. The combined dataset from these sources comprises 70,083 binding CDR3β-peptide pairs and 2,689,709 non-binding CDR3β-peptide pairs. The 10X dataset included both binding and non-binding samples, with a very small proportion of binding pairs, accounting for less than 1% of the total data.

To create additional non-binding data to increase the diversity of training set, we proposed a strategy for data generation which can be done via two approaches. The first approach involved extracting representative sequences from three publicly available neoantigen datasets—namely, TSNAdb, Neodb, and NEPdb—and randomly combining them with CDR3β sequences from previously collected data (from McPAS-TCR, TBAdb, VDJdb, IEDB, and 10X). This resulted in 174,944 CDR3β-peptide pairs with 2,506 unique peptides in the non-binding dataset. In the second approach, we used 71 random CDR3β sequences from tumor infiltrating lymphocytes (TIL)[7] coupled with peptides from public wild-type datasets (McPAS-TCR, TBAdb, VDJdb, IEDB, and 10X), resulting in 132,979 CDR3β-peptide pairs. These data generation strategies were employed throughout the entire manuscript, including further model evaluations to ensure the non-binding label of the synthesized data.

## Data organization for model training and testing

The training data affects the learning of the models. Therefore, we first focused on the amount of data needed for model training. From the total 3,647 unique peptides related to interactions gathered in our dataset, we tried to find out the appropriate amount of training data to stabilize the model’s performance. Our previous work showed that the model’s performance relied significantly on the number of unique peptides. Consequently, we chose an increasing number of peptides and their corresponding interactions for training, specifically 1,000, 1,200, 1,400, 1,600, 1,800, 2,000, 2,400, 2,600, 2,800, 3,000, and 3,200, then performed a 10-fold cross validation training strategy to find the best amount of training data needed for the model generalization. The training peptides were selected based on their frequency (the number of interactions where they appeared), as we prioritized learning from interactions that are common in real life. By gradually increasing the number of peptides, we aimed to strike a balance between data complexity and model generalization. We assessed the model’s robustness using the mean AUC across 10 validation folds (Supplementary Figure S2).

Processing of CDR3β and peptide sequences for model input

Figure 1B briefly described the data preprocessing steps preparing input for the knowledge distillation model. In those steps, the processing from dihedral angles by the Biopython library to the input matrix was simplified. This data processing is further described in details in Supplementary Figure S1. Indeed, the phi angles and psi angles of CDR3β and peptides obtained OmegaFold and Biopython are of length $l-2$, with $l$ is the length of the CDR3β/peptide amino acid sequence, excluding the first and last amino acids. To ensure uniform input dimensions for the learning models, the matrices are then padded by zeros to 17x2 for CDR3β and 9x2 for the peptide, aligning with the maximum amino acid sequence lengths observed in the dataset (maximum length of 19 for CDR3β amino acid sequences, and maximum length of 11 for peptide amino acid sequences). Finally, these padded matrices are concatenated into a single 17x4 matrix, integrating both sequence and structural information, which serves as the input for the model.

## epiTCR-KDA model training

## epiTCR-KDA model is a knowledge distillation model, which was designed in comparison with a list of machine learning models using the dihedral angles of CDR3β and peptide as input (Supplementary Table S3).

## The epiTCR-KDA structure began with the transformation of input CDR3β and peptide sequences into matrices of phi and psi angles. These matrices were then concatenated and zero-padded to form a 17x4 matrix. In this matrix, 17 rows represented the dimension obtained from the longest sequence, and 4 columns represented two pairs of phi and psi angles from both the CDR3β and peptide.

## Using this matrix as input, both the student and teacher models were built based on convolutional neural networks (CNNs). The teacher model was designed for binary classification. It started with a convolutional layer of 64 filters of size 3x3 (with a stride of (2, 2)), followed by a LeakyReLU activation (α = 0.2), and a MaxPooling2D layer with a 2x2 filter and stride = 1. Subsequently, two convolutional layers with 128 and 256 filters (using the same filter size and stride) were applied. The output from the last layer was flattened into a 1D vector, followed by a fully connected layer and a single unit equipped with sigmoid activation for binary classification.

The student model replicated the teacher’s predictions with reduced complexity by reusing three convolutional layers with 16, 32, and 64 filters, respectively, while keeping other layers unchanged from the teacher model. The distillation process involved a Distiller object containing both models. During training, the Distiller object was compiled using Adam optimizer, with BinaryAccuracy metric for evaluation, BinaryCrossentropy loss function for the student, and KLDivergence for distillation loss evaluation. The ‘alpha’ was set at 0.1 to balance the hard student predictions and the soft distillation loss from the teacher. The ‘temperature’ was adjusted to 10 to moderate the teacher’s predicted probabilities before transferring them to the student. The chosen batch size, set at 64, determined the quantity of training samples processed in each iteration, potentially increasing the training speed and model convergence. These parameters were crucial for achieving effective model performance during training.

## Retraining public models

NetTCR, epiTCR, and TEINet was retrained based on the code availability. In general, these models were retrained using the same code provided by the authors, with the same training set as epiTCR-KDA’s.

For NetTCR, the input data comprised only the CDR3β and peptide sequences, which were encoded using the BLOSUM50 matrix. These sequences were then processed through 1D convolutional layers with multiple kernel sizes to capture diverse sequence patterns. The model was retrained using a nested 5-fold cross-validation approach, with training capped at 300 epochs and early stopping triggered after 50 epochs without improvement. The Adam optimizer, with a learning rate of 0.001, was utilized alongside binary cross-entropy as the loss function. A batch size of 128 was maintained throughout the training process. After applying max-pooling, the resulting features were concatenated and fed into fully connected layers for final predictions. Early stopping mitigated overfitting, leading to improved model generalization on unseen data.

epiTCR was retrained using the Random Forest algorithm from scikit-learn to tackle TCR-peptide binding classification. CDR3β sequences (8–19 amino acids) and peptide sequences (8–11 amino acids) were encoded with BLOSUM62, and zero-padding was applied for shorter sequences. The sequences were flattened into a 600-feature vector. Binding and non-binding interactions were labeled as 1 and 0, respectively. The retrained model was evaluated using five-fold cross-validation and provided probability-based predictions for new TCR-peptide pairs, using a default threshold of 0.5.

TEINet was retrained using Python 3.6 and the PyTorch framework, with evaluation conducted through 5-fold cross-validation. In each fold, positive data were split into training and validation sets, while negative samples were dynamically generated during training to prevent data leakage. Negative pairs were sampled at a 10:1 ratio relative to positive pairs. The retraining process minimized binary cross-entropy loss using the Adam optimizer, with an initial learning rate of 1 × 10⁻³. The model was trained for 50 epochs with a batch size of 48, and the learning rate was reduced by a factor of 0.1 at the 21st and 27th epochs.

## General model evaluation on remaining data from training

## Our initial evaluation of the model’s performance focused on the remaining data that had not been utilized during model training. We assessed the model’s performance using the Area Under the Curve (AUC) across four scenarios:

## All CDR3β-peptide pairs: all interactions had not appeared during the training process.

## Interactions of seen peptides: interactions involving peptides it had encountered during training.

## Interactions of unseen peptides: interactions with peptides it had not seen during training.

## Interactions of seven dominant unseen peptides: interactions involving seven specific unseen peptides that were prevalent in the entire dataset.

In each scenarios, we evaluated the public original models (including epiTCR, NetTCR, BERTRand, TEIM-Seq, TEINet, and ImRex) and some retrained models (including epiTCR, NetTCR, and TEINet) in case of training code published by the authors. The training sets were different across original models, thus the seen and unseen peptides from training change accordingly to the specific model (Supplementary Table S4, Supplementary Figure S3, S4). Additionally, the groups of seen peptides and unseen peptides in all model training sets were used for the fair comparison ( Figure 2A, Supplementary Table S4, S5).

## Testing epiTCR-KDA generalization

Beside epiTCR-KDA’s model performance evaluated on data seen/unseen in training and evaluated on different datasets, we also evaluated the model generalization by its performance predicting the interactions of which peptides were derived from different pathogen sources.

The pathogen source was indicated under different column names in different databases, specifically, column “Pathology”, “Organisms”, and “Epitope species” in McPAS-TCR, IEDB, and VDJdb, respectively. In TBAdb, this information was inferred from the “Disease name” and “Category” column. The detailed pathogen annotations were then converted into major groups based on their kingdoms (ie. virus, human, and other).

The AUC, accuracy, sensitivity, and specificity was reported for each group of pathogens, with seen and unseen interactions in Supplementary Figure S5.

## Evaluating the impact of training data on model’s prediction

The process of learning from CDR3β and peptide sequences significantly influenced the model's predictions. To do this, we explored whether the similarity between training and testing CDR3β/peptide sequences affected the model's predictions. Therefore, we compared the predicted interactions of CDR3β/peptides in the testing set with learned interaction labels of similar CDR3β/peptides.

The method thereby described was applied for peptides, and the same calculations were also applied for CDR3β.

Supposing the phi and psi angles of any two peptides were:

$${peptide}_{i}=\left\{ \phi_{i}, \psi_{i} \right\}=\{\phi_{i1}, \phi_{i2}, \ldots, \phi_{in-2}, \psi_{i1}, \psi_{i2}, \ldots, \psi_{in-2}\}=\{x_{1},x_{2},\ldots,x_{2n-4}\}$$

$${peptide}_{j} =\left\{ \phi_{j}, \psi_{j} \right\}=\{\phi_{j1}, \phi_{j2}, \ldots, \phi_{jn-2}, \psi_{j1}, \psi_{j2}, \ldots, \psi_{jn-2}\}=\left\{ y_{1}{,y}_{2},\ldots,y_{2n-4} \right\},$$

with n is the length of peptide sequences.

The similarity between any two peptides relied on the cosine similarity between the phi and psi angles of those peptides. The cosine similarity was defined as:

$${cosine\_similarity}_{\left( {peptide}_{i}, {peptide}_{j} \right)}=\frac{\sum_{1}^{2n-4} x_{i}y_{j}}{\sqrt{\sum_{1}^{2n-4} x_{i}^{2}}\sqrt{\sum_{1}^{2n-4} y_{j}^{2}}}(1)$$

given that -1 $\leq$ $cosine\_similarity\left( {peptide}_{i}, {peptide}_{j} \right)$ $\leq$ 1.

$RMSE=$ $\sqrt{\frac{{({\%}_{pos\_test} - {\%}_{pos\_train})}^{2} + {({\%}_{neg\_test} - {\%}_{neg\_train})}^{2}}{2}}$ (2)

We selected nine representative CDR3β and nine peptides, each representing distinct groups of CDR3β and peptides available in the testing sets. These groups were formed based on the cosine similarity between CDR3β dihedral angles and/or peptide dihedral angles (equation (1)). For each representative CDR3β/peptide, we organized trained CDR3β/peptides into bins based on their cosine similarity to the representative. The number of CDR3β/peptides having cosine similarity below 0.5 was too small, so we only reported the group of CDR3β/peptides having cosine similarity from 0.5 and above. We categorized training CDR3β/peptides into five levels of similarities: (0.5 – 0.59), (0.6 – 0.69), (0.7 – 0.79), (0.8 – 0.89), and (0.9 – 0.99). We then calculated the Root Mean Square Error (RMSE) reflecting the difference between the labels (binding/non-binding) of CDR3β-peptide pairs related to CDR3β/peptides in the bins and the model’s prediction on interactions of CDR3β/peptides under consideration (equation (2)).

## epiTCR-KDA performance on different testing scenarios

To assess the model’s generalizability, we conducted benchmark evaluations of epiTCR-KDA, public original models, and retrained models on distinct datasets sourced from other research papers. This evaluation occurred in two scenarios.

First, we gathered data from Panpep[8] and catELMo[9]. These datasets exclusively contained binding pairs, necessitating the generation of non-binding pairs using the data generation strategies previously employed for the training and testing sets of our models. The first non-binding dataset comprised 313,161 CDR3β-peptide pairs, consisting of CDR3β sequences from TIL[10] and public wild-type peptides from TSNAdb[11], Neodb[12], and NEPdb[13] (labeled as (1)). The second non-binding dataset included 402,732 pairs from wild-type peptides with public CDR3β sequences (labeled as (2)). Additionally, we utilized binding data from Panpep (10,397 pairs, labeled as (3)) and catELMo (85,020 pairs, labeled as (4)). By exhaustively combining these datasets, we generated a total of nine testing sets (as detailed in Supplementary Table S7). This approach ensured that model evaluation remained independent of any single data source.

# In the second scenario, we collected a COVID-19 dataset [14], which consisted of 2,120,140 CDR3β-peptide pairs. Among these, there were 2,120,100 non-binding pairs and only 40 binding CDR3β-peptide pairs. We employed this dataset for independent testing because the peptides and all related TCRs originated from a context vastly different from the pairs that had been trained and tested by our models.

# In the subsequent experiment, we investigated whether the models’ performance was affected by the data composition, particularly when we altered the number of non-binding pairs and the amount of unseen peptide interactions. Using the two binding datasets collected from Panpep and catELMo, along with the two non-binding sets generated by our strategies, we adjusted the ratios of binding to non-binding data and the ratios of seen to unseen peptide interactions. Specifically, we created four testing sets with the number of non-binding pairs equal to, double, triple, and quadruple the number of binding CDR3β-peptide pairs. Additionally, we constructed four other CDR3β-peptide sets, each containing the number of unseen peptides equal to five, ten, and twenty times the number of peptides seen in the training set (as detailed in Supplementary Table S8). These data settings were designed to reflect real-life scenarios, where unseen peptide interactions and non-binding interactions significantly contribute to the prediction set.

# For all above experiments, the Area Under the Curve (AUC) was reported for the tools participating in the evaluation.

## Runtime evaluation

To evaluate the scability of epiTCR-KDA and other TCR-peptide binding predictors, we randomly generated datasets with increasing sizes from 2,000 to 1,000,000 TCR-peptide pairs. epiTCR-KDA, ATM-TCR, BERTrand, TEIM-seq, and TEINet required GPU resources and were executed on machines equipped with NVIDIA RTX A5000 GPUs (24GB GDDR6, 384-bit) and 100GB of RAM, whereas epiTCR and NetTCR were run on Intel Xeon Silver 4310 CPUs (18M, 2.10 GHz) with the same RAM configuration. epiTCR-KDA was tested twice, with and without 3D structure simulation from CDR3b/peptide amino acid sequences using OmegaFold, for a comprehensive benchmark on the runtime of the knowledge distillation model and the whole process. In reality, all required 3D structures can be run at once and separately from the knowledge distillation model by any of the publicly available protein spatial structure predictors (for example: AlphaFold2 and ESMFold). Here we use OmegaFold for our 3D structure simulation because of its scalability. For all CDR3b and peptide amino acid sequences used in this work, we have deposed a library of all required 3D structures, which can be extended for further usage of epiTCR-KDA.

# Supplementary Tables

Table S1. The number of observation in five datasets.

| Datasets | Data counts | Date of collection | Link to dataset |
| --- | --- | --- | --- |
| TBAdb | 1,015 | June 16th, 2022 | https://gitlab.com/immunomind/immunarch/blob/master/private/TBAdb.xlsx |
| VDJdb | 7,581 | June 16th, 2022 | https://vdjdb.cdr3.net/search |
| IEDB | 51,680 | June 16th, 2022 | https://www.iedb.org |
| McPAS-TCR | 3,646 | August 5th, 2022 | http://friedmanlab.weizmann.ac.il/McPAS-TCR |
| 10X | 2,689,720 | June 20th, 2022 | 1. <https://www.10xgenomics.com/resources/datasets/cd-8-plus-t-cells-of-healthy-donor-1-1-standard-3-0-2> 2. <https://www.10xgenomics.com/resources/datasets/cd-8-plus-t-cells-of-healthy-donor-2-1-standard-3-0-2> 3. <https://www.10xgenomics.com/resources/datasets/cd-8-plus-t-cells-of-healthy-donor-3-1-standard-3-0-2> 4. <https://www.10xgenomics.com/resources/datasets/cd-8-plus-t-cells-of-healthy-donor-4-1-standard-3-0-2> |
| Total | 2,753,642 |  |  |

Table S2. A table detailing the specific statistics of the collected data and the data generated for the training and testing used for benchmarking epiTCR-KDA and retrained models.

| Number of … | | in training set | in testing set |
| --- | --- | --- | --- |
| CDR3β-peptide pairs | Binding pairs | 34380 | 29553 |
|  | Non-binding pairs | 1598234 | 1399398 |
|  | Total | 1632614 | 1428951 |
| Collected CDR3β-peptide pairs | Binding pairs | 34380 | 29553 |
|  | Non-binding pairs | 1463832 | 1225877 |
|  | Total | 1498212 | 1255430 |
| Generated CDR3β-peptide pairs (non-binding only) | | 134402 | 173521 |
| Unique peptides | Unique seen peptides | NA | 1948 |
|  | Unique unseen peptides | NA | 1641 |
|  | Collected unique peptides | 159 | 1174 |
|  | Generated unique peptides | 1894 | 2444 |
|  | Total | 2,000 | 3589 |
| Unique CDR3β | Unique seen CDR3β | NA | 63237 |
|  | Unique unseen CDR3β | NA | 26613 |
|  | Collected unique CDR3β | 87375 | 89828 |
|  | Generated unique CDR3β | 71 | 71 |
|  | Total | 94725 | 89850 |

Table S3. Different model’s performance applied on dihedral angle (DA) information.

| Method | Set | Performance metrics | | | |  | Set | Performance metrics | | | |
| --- | --- | --- | --- | --- | --- | --- | --- | --- | --- | --- | --- |
| - | - | AUC | Accuracy | Sensitivity | Specificity | - | - | AUC | Accuracy | Sensitivity | Specificity |
| epiTCR-KDA | Overall | 0.984 | 0.931 | 0.964 | 0.93 | MLP | Overall | 0.984 | 0.931 | 0.964 | 0.93 |
|  | Seen | 0.998 | 0.969 | 0.992 | 0.968 |  | Seen | 0.998 | 0.969 | 0.992 | 0.968 |
|  | Unseen | 0.913 | 0.906 | 0.833 | 0.906 |  | Unseen | 0.913 | 0.906 | 0.833 | 0.906 |
| Naïve Bayes | Overall | 0.599 | 0.87 | 0.396 | 0.881 | CNN* | Overall | 0.599 | 0.87 | 0.396 | 0.881 |
|  | Seen | 0.669 | 0.864 | 0.424 | 0.884 |  | Seen | 0.669 | 0.864 | 0.424 | 0.884 |
|  | Unseen | 0.496 | 0.874 | 0.267 | 0.878 |  | Unseen | 0.496 | 0.874 | 0.267 | 0.878 |
| KNN | Overall | 0.818 | 0.701 | 0.773 | 0.699 | LSTM | Overall | 0.818 | 0.70 | 0.773 | 0.699 |
|  | Seen | 0.892 | 0.818 | 0.845 | 0.817 |  | Seen | 0.892 | 0.818 | 0.845 | 0.817 |
|  | Unseen | 0.537 | 0.624 | 0.439 | 0.625 |  | Unseen | 0.537 | 0.624 | 0.439 | 0.625 |
| Logictic Regrestion | Overall | 0.688 | 0.684 | 0.651 | 0.685 | Bi  LSTM | Overall | 0.688 | 0.684 | 0.651 | 0.685 |
|  | Seen | 0.746 | 0.677 | 0.694 | 0.676 |  | Seen | 0.746 | 0.677 | 0.694 | 0.676 |
|  | Unseen | 0.551 | 0.688 | 0.448 | 0.69 |  | Unseen | 0.551 | 0.688 | 0.448 | 0.69 |
| Random Forest | Overall | 0.915 | 0.952 | 0.811 | 0.955 | RNN | Overall | 0.915 | 0.952 | 0.811 | 0.955 |
|  | Seen | 0.951 | 0.888 | 0.984 | 0.884 |  | Seen | 0.951 | 0.888 | 0.984 | 0.884 |
|  | Unseen | 0.537 | 0.994 | 0.002 | 1 |  | Unseen | 0.537 | 0.994 | 0.002 | 1 |
| XGBoost | Overall | 0.624 | 0.861 | 0.35 | 0.872 | Transformer | Overall | 0.624 | 0.861 | 0.35 | 0.872 |
|  | Seen | 0.722 | 0.893 | 0.381 | 0.916 |  | Seen | 0.722 | 0.893 | 0.381 | 0.916 |
|  | Unseen | 0.477 | 0.841 | 0.205 | 0.845 |  | Unseen | 0.477 | 0.841 | 0.205 | 0.845 |

Notes: The models include Naive Bayes, K-Nearest Neighbors (KNN), Logistic Regression, Random Forest, XGBoost, Multi-Layer Perceptron (MLP), Convolutional Neural Network (CNN), Long Short-Term Memory (LSTM), Bidirectional Long Short-Term Memory (BiLSTM), Recurrent Neural Network (RNN), and Transformer.

* CNN was a linear combination of the epiTCR-KDA’s Teacher model and Student model, in which the Teacher model learned from the input data and fed its output to the Student model.

Table S4. A table detailing counts of CDR3β-peptide pairs in testing used for benchmarking epiTCR-KDA and original models.

|  | N of binding pairs | | N of non-binding pairs | | N of peptides | |
| --- | --- | --- | --- | --- | --- | --- |
|  | seen | unseen | seen | unseen | seen | unseen |
| epiTCR-KDA | 24362 | 5191 | 542346 | 857052 | 1948 | 1641 |
| epiTCR | 27235 | 2318 | 1165169 | 234229 | 442 | 3147 |
| NetTCR | 43 | 29510 | 50058 | 1349340 | 4 | 3585 |
| BERTrand | 9883 | 19670 | 828742 | 570656 | 293 | 3296 |
| TEIM-seq | 20679 | 8874 | 362729 | 1036669 | 273 | 3316 |
| TEInet | 10792 | 18761 | 763865 | 635533 | 195 | 3394 |
| ImRex | 48595 | 11612 | 961248 | 407496 | 382 | 3207 |

Table S5. Benchmark result of epiTCR-KDA and other original models on the overlapping seen (36,260 TCR-peptide pairs) and overlapping unseen data (41,482 TCR-peptide pairs). The performance metrics could not be calculated for epiTCR and NetTCR because epiTCR predicted all pairs as binding while NetTCR predicted all pairs as non-binding, as coherent with benchmarked results shown in Figure 2A.

| Original models | Overlapping set | Performance metrics | | | |
| --- | --- | --- | --- | --- | --- |
| - | - | AUC | Accuracy | Sensitivity | Specificity |
| epiTCR-KDA | Seen | 1 | 1 | 1 | 1 |
| epiTCR-KDA | Unseen | 0.856 | 0.865 | 0.802 | 0.866 |
| epiTCR | Seen | 0.867 | 1 | 0.25 | 1 |
| epiTCR | Unseen | - | - | - | - |
| NetTCR | Seen | - | - | - | - |
| NetTCR | Unseen | - | - | - | - |
| BERTrand | Seen | - | - | - | - |
| BERTrand | Unseen | 0.483 | 0.687 | 0.274 | 0.688 |
| TEIM-seq | Seen | 0.619 | 0.265 | 1 | 0.265 |
| TEIM-seq | Unseen | 0.62 | 0.324 | 0.745 | 0.323 |
| TEINet | Seen | 0.567 | 0.95 | 0 | 0.95 |
| TEINet | Unseen | 0.03 | 0.46 | 0.019 | 0.462 |
| ImRex | Seen | 0.463 | 0.648 | 0.5 | 0.648 |
| ImRex | Unseen | 0.47 | 0.54 | 0.387 | 0.541 |

Table S6. Counts of CDR3β-peptide pairs corresponding to different pathogens

|  |  | Binding | Non-binding |
| --- | --- | --- | --- |
| Training set | Virus | 33,516 | 699,704 |
|  | Human | 851 | 647,847 |
|  | Other | 13 | 250,683 |
| Test set | Virus | 27,808 | 800,788 |
|  | Human | 1,216 | 348,516 |
|  | Other | 478 | 250,094 |

Table S7. List number of pairs derived from each of the 7 dominant peptides

| No | Peptide | n_pairs |
| --- | --- | --- |
| 1 | GLCTLVAML | 65,713 |
| 2 | NLVPMVATV | 65,109 |
| 3 | GILGFVFTL | 64,111 |
| 4 | TPRVTGGGAM | 63,959 |
| 5 | ELAGIGILTV | 63,495 |
| 6 | AVFDRKSDAK | 62,774 |
| 7 | KLGGALQAK | 62,237 |

Table S8. Counts of CDR3β-peptide pairs in different testing sets used in Figure 4A and 4B

| No | Dataset | n_obs | n_pos_  obs | n_neg_  obs | n_unique_pep | n_unseen_pep | n_seen_pep | n_unique_tcr | tcr_unseen | tcr_seen |
| --- | --- | --- | --- | --- | --- | --- | --- | --- | --- | --- |
| 1 | (1)(3) | 398181 | 85020 | 313161 | 3540 | 1756 | 1784 | 81780 | 81777 | 3 |
| 2 | (1)(4) | 323558 | 10397 | 313161 | 3014 | 1286 | 1728 | 10416 | 10416 | 0 |
| 3 | (2)(3) | 487752 | 85020 | 402732 | 3540 | 1756 | 1784 | 81694 | 81691 | 3 |
| 4 | (2)(4) | 413129 | 10397 | 402732 | 3014 | 1286 | 1728 | 10276 | 10276 | 0 |
| 5 | (1)(3)(4) | 408578 | 95417 | 313161 | 3553 | 1764 | 1789 | 86743 | 86740 | 3 |
| 6 | (2)(3)(4) | 498149 | 95417 | 402732 | 3553 | 1764 | 1789 | 86656 | 86653 | 3 |
| 7 | (1)(2)(3) | 800913 | 85020 | 715893 | 3540 | 1756 | 1784 | 81858 | 81855 | 3 |
| 8 | (1)(2)(4) | 726290 | 10397 | 715893 | 3014 | 1286 | 1728 | 10544 | 10544 | 0 |
| 9 | (1)(2)(3)(4) | 811310 | 95417 | 715893 | 3553 | 1764 | 1789 | 86815 | 86812 | 3 |
| 10 | COVID | 2120140 | 40 | 2120100 | 1265 | 1255 | 10 | 1489 | 1442 | 47 |

Note:

(1): non-binding pairs made of CDR3β from TIL[10] and wildtype peptides,

(2): non-binding pairs made of publicly collected CDR3β[1, 2, 3, 4, 5] and wildtype peptides[11, 12, 13],

(3): binding pairs from Panpep[8],

(4): binding pairs from catELMo[9]

Table S9. Counts of CDR3β-peptide pairs in different testing sets used in Figure 4C

| No | Dataset | n_obs | n_pos_obs | n_neg_  obs | n_unique_pep | n_unseen_pep | n_seen_pep | n_unique_tcr | n_unseen_tcr | n_seen_tcr |
| --- | --- | --- | --- | --- | --- | --- | --- | --- | --- | --- |
| 1 | n.neg = n.pos | 190834 | 95417 | 95417 | 3553 | 1764 | 1789 | 86815 | 86812 | 3 |
| 2 | n.neg = 2n.pos | 286251 | 95417 | 190834 | 3553 | 1764 | 1789 | 86815 | 86812 | 3 |
| 3 | n.neg = 3n.pos | 381668 | 95417 | 286251 | 3553 | 1764 | 1789 | 86815 | 86812 | 3 |
| 4 | n.neg = 4n.pos | 477085 | 95417 | 381668 | 3553 | 1764 | 1789 | 86815 | 86812 | 3 |

# Supplementary Figures


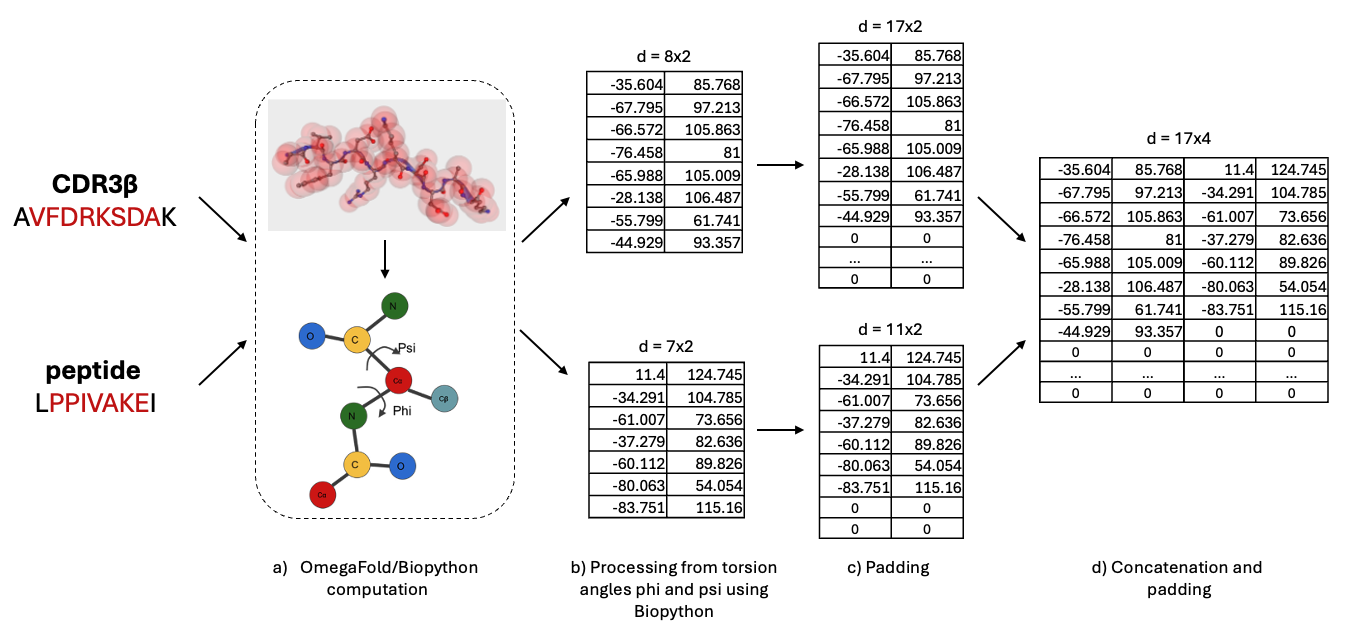


Figure S1. Detailed description of input data preprocessing. CDR3β and peptide amino acid sequences were preprocessed individually, starting from the sequence conversion to 3D structures via OmegaFold, followed by the dihedral angle calculation by Biopython, zero-padding to the obtain the same matrix dimensions as the ones of the longest CDR3β or peptide sequences, and lastly the concatenation of the CDR3β or peptide representation matrices. The output matrix of this process is then served as the input of the knowledge distillation learning model.


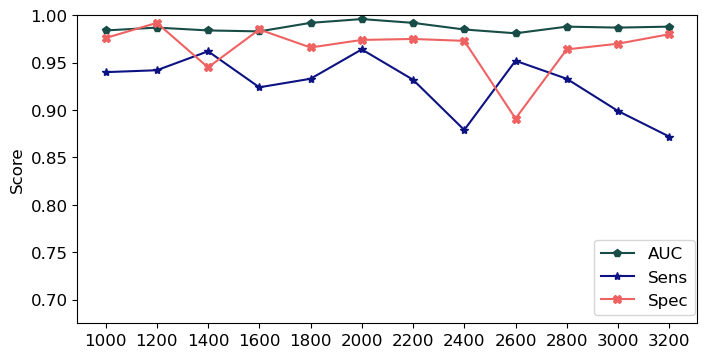


Figure S2. Model performance across different amount of training data, starting from interactions of 1,000 peptides to 3,200 peptides. The reported values were the mean model performance across 10 folds of training validation.

Our testing on gradual changing amount of training data (Supplementary Figure S2) showed that the top 2,000 peptides led to the good performance in AUC. Furthermore, the model trained with 2,000 peptides had very small trade-off between the model sensitivity and specificity. Therefore, the model trained on this amount of training data was expected to have robust prediction.


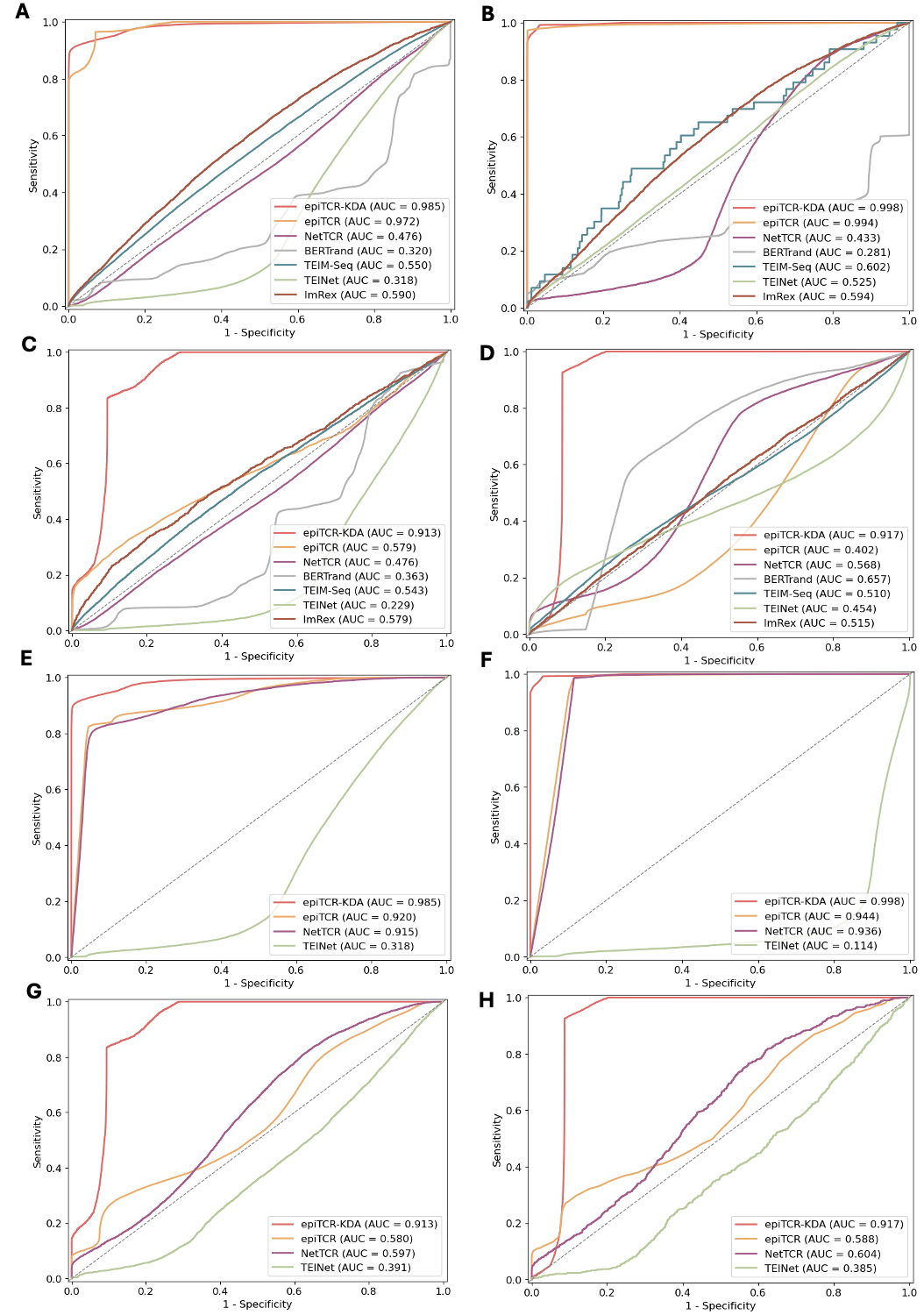


Figure S3. The prediction AUC of epiTCR-KDA, original models (including epiTCR, NetTCR, BERTrand, TEIM-Seq, TEINet, and ImRex), and retrained models (including epiTCR, NetTCR, and TEINet) on ten overall testing sets (data remaining from training set), with four benchmark settings: (A) on overall interactions with original models, (B) on interactions of seen peptides with original models, (C) on interactions of unseen peptides with original models, (D) on interactions of seven dominant unseen peptides with original models, (E) on overall interactions with retrained models, (F) on interactions of seen peptides with retrained models, (G) on interactions of unseen peptides with retrained models, and (H) on interactions of seven dominant unseen peptides with retrained models.


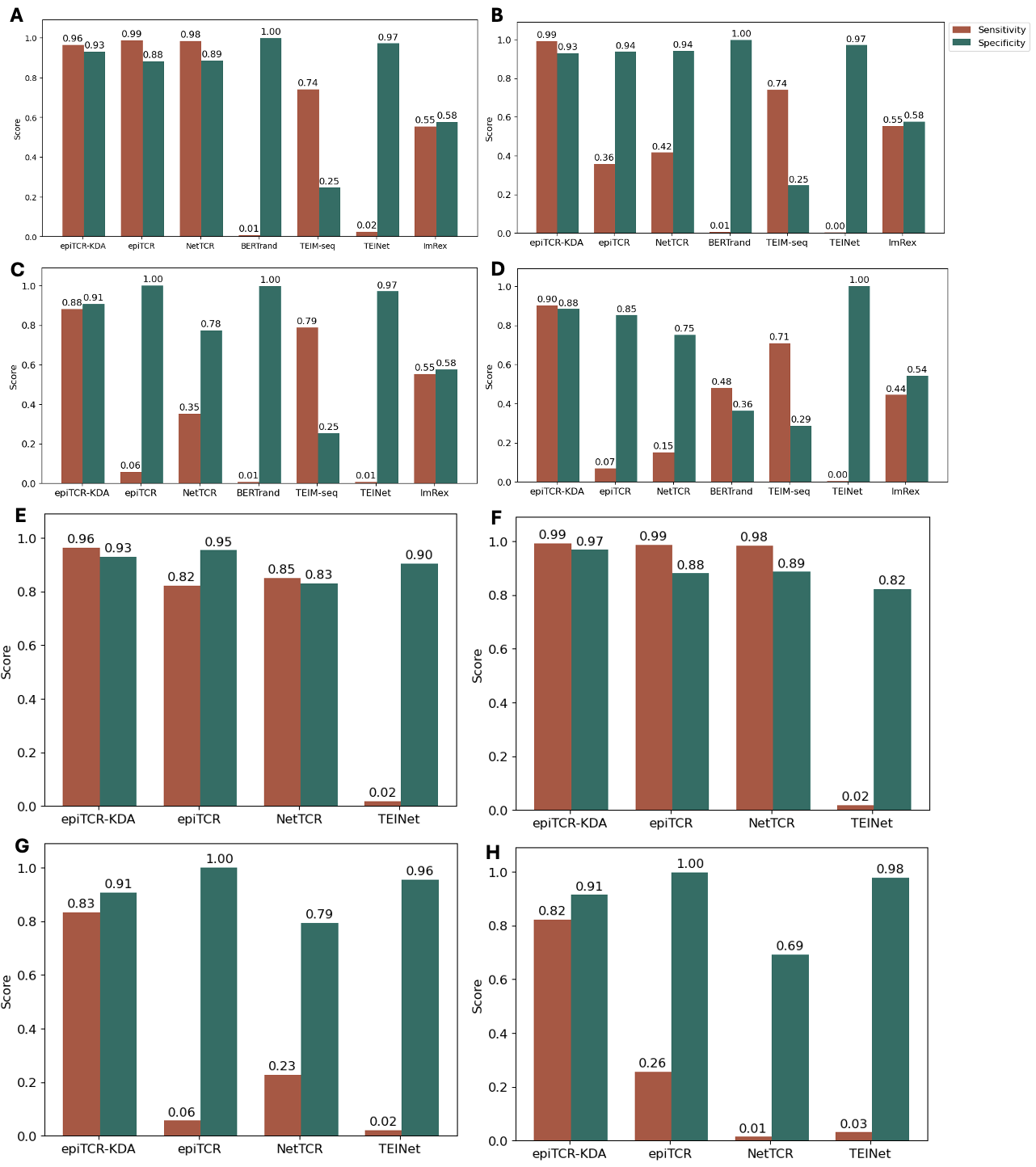


Figure S4. The sensitivity and specificity of the epiTCR-KDA, original models (including epiTCR, NetTCR, BERTrand, TEIM-Seq, TEINet, and ImRex), and retrained models (including epiTCR, NetTCR, TEINet) on ten overall testing sets (data remaining from training set), on four benchmark settings: (A) on overall interactions with original models, (B) on interactions of seen peptides with original models, (C) on interactions of unseen peptides with original models, (D) on interactions of seven dominant unseen peptides with original models, (E) on overall interactions with retrained models, (F) on interactions of seen peptides with retrained models, (G) on interactions of unseen peptides with retrained models, and (H) on interactions of seven dominant unseen peptides with retrained models.


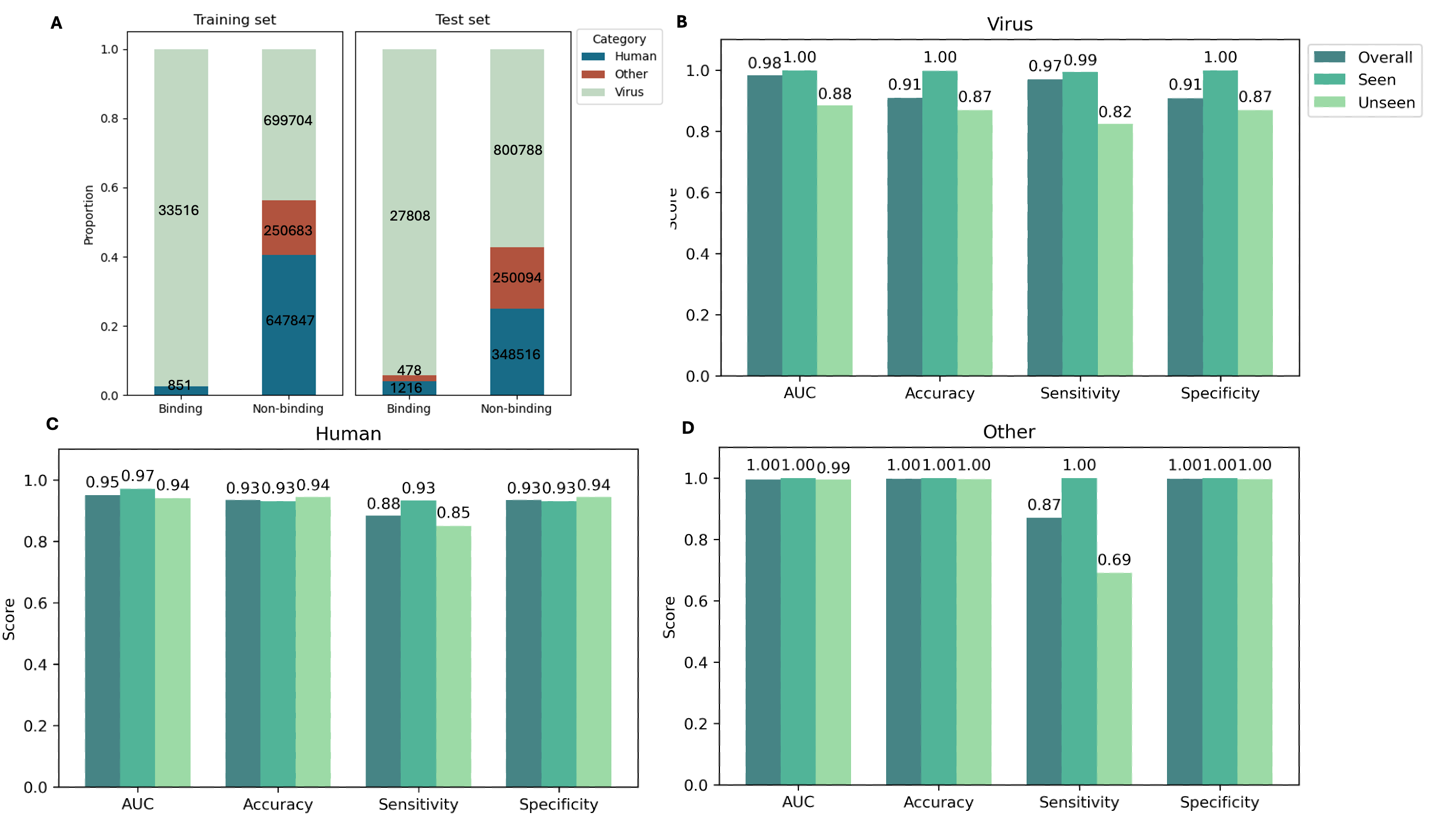


Figure S5. The number of TCR-peptide pairs in training data and testing data corresponding to different pathogen sources: (B) virus, (C) human, and (D) others, and epiTCR-KDA’s performance across those sources.

Supplementary Figure S5A summarized the binding and non-binding TCR-peptide pairs in which the peptides derived from virus, human, and other pathogen sources. The data summary revealed a significant data imbalance between the number of binding and non-binding pairs, in which the non-binding pairs dominated across all considered pathogen categories. Particularly, the interactions of peptides derived from other pathogens, were absent from the training data, suggesting potential challenges for model training.

Supplementary Figure S5B-C-D described epiTCR-KDA performance on interactions of different pathogen sources. Particularly, epiTCR-KDA exhibited robust AUC, accuracy, and specificity (each metrics of around 0.93 – 1) in predicting interactions of peptides derived from both human and other pathogens (Supplementary Figure S5C, S5D). Notably, the interactions of peptides from other pathogen sources were predicted with slightly higher performance, with AUC, accuracy, and specificity around 1. On the other hand, the prediction sensitivity on interactions of those pathogen sources dropped, with a dramatic change on the interactions of unseen peptides derived from other pathogens. This phenomenon might be because of the low amount of training data derived from those pathogens.

For the interactions of peptides derived from virus (Supplementary Figure S5B), epiTCR-KDA exhibited high performance on overall and interactions of peptides seen during training. However, the performance metrics notably dropped for the interactions of peptides unseen during training. This notable decrease in performance is inline with the model’s performance on the set of data derived from dominant and challenging peptides (Figure 2E).

##
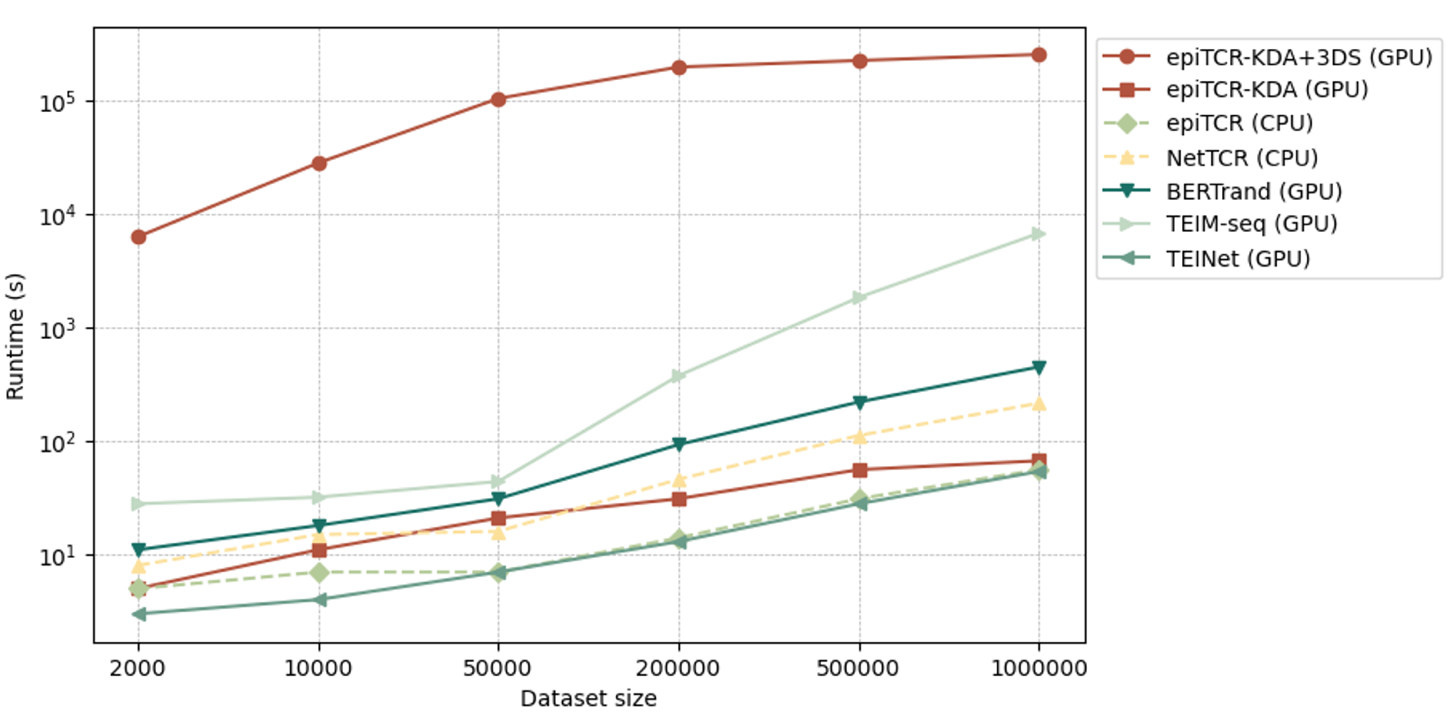


Figure S6. The runtime of epiTCR-KDA (with and without 3D structure simulation), epiTCR, ATM-TCR, NetTCR, BERTrand, TEIM-seq, and TEINet on generated datasets of sizes from 2,000 – 1,000,000 TCR-peptide pairs.

## epiTCR-KDA with 3D structure simulation required the longest runtime (approximate 3 days on the largest dataset, with each 3D structure prediction taking about three seconds), while epiTCR-KDA with prepared 3D structures needed relatively small amount of time, approximate 100 seconds to finish predicting the largest dataset. This result highlighed that the knowledge distillation model of epiTCR-KDA had good scalability. The most time-consuming task of epiTCR-KDA involved the spatial structure simulation of TCR and peptides, which was not the main optimisation of the tool.

## The other benchmarked tools also gave prediction within a reasonable amount of time.

# References

[1] N. Tickotsky, T. Sagiv, J. Prilusky, E. Shifrut, and N. Friedman, “McPAS-TCR: a manually curated catalogue of pathology-associated T cell receptor sequences,” *Bioinformatics*, vol. 33, no. 18, pp. 2924–2929, Sep. 2017, doi: 10.1093/BIOINFORMATICS/BTX286.

[2] W. Zhang *et al.*, “PIRD: Pan Immune Repertoire Database,” *Bioinformatics*, vol. 36, no. 3, pp. 897–903, Feb. 2020, doi: 10.1093/BIOINFORMATICS/BTZ614.

[3] M. Shugay *et al.*, “VDJdb: a curated database of T-cell receptor sequences with known antigen specificity,” *Nucleic Acids Res*, vol. 46, no. D1, pp. D419–D427, Jan. 2018, doi: 10.1093/NAR/GKX760.

[4] R. Vita *et al.*, “The Immune Epitope Database (IEDB): 2018 update,” *Nucleic Acids Res*, vol. 47, no. D1, pp. D339–D343, Jan. 2019, doi: 10.1093/NAR/GKY1006.

[5] “A New Way of Exploring Immunity - Linking Highly Multiplexed Antigen Recognition to Immune Repertoire and Phenotype | Technology Networks.” Accessed: Mar. 22, 2024. [Online]. Available: https://www.technologynetworks.com/immunology/application-notes/a-new-way-of-exploring-immunity-linking-highly-multiplexed-antigen-recognition-to-immune-repertoire-332554

[6] A. Montemurro *et al.*, “NetTCR-2.0 enables accurate prediction of TCR-peptide binding by using paired TCRα and β sequence data,” *Commun Biol*, vol. 4, no. 1, p. 1060, 2021, doi: 10.1038/s42003-021-02610-3.

[7] T. M. Q. Pham *et al.*, “The T Cell Receptor β Chain Repertoire of Tumor Infiltrating Lymphocytes Improves Neoantigen Prediction and Prioritization,” *Elife*, vol. 13, 2024, doi: 10.7554/ELIFE.94658.1.

[8] Y. Gao *et al.*, “Pan-Peptide Meta Learning for T-cell receptor–antigen binding recognition,” *Nature Machine Intelligence 2023 5:3*, vol. 5, no. 3, pp. 236–249, Mar. 2023, doi: 10.1038/s42256-023-00619-3.

[9] P. Zhang, S. Bang, M. Cai, and H. Lee, “Context-Aware Amino Acid Embedding Advances Analysis of TCR-Epitope Interactions,” Apr. 2023, doi: 10.1101/2023.04.12.536635.

[10] T. Mong *et al.*, “The T Cell Receptor β Chain Repertoire of Tumor Infiltrating Lymphocytes Improves Neoantigen Prediction and Prioritization,” *bioRxiv*, p. 2023.11.16.567478, Nov. 2023, doi: 10.1101/2023.11.16.567478.

[11] J. Wu *et al.*, “TSNAdb v2.0: The Updated Version of Tumor-Specific Neoantigen Database,” *Genomics Proteomics Bioinformatics*, vol. 21, no. 2, pp. 259–266, Apr. 2023, doi: 10.1016/j.gpb.2022.09.012.

[12] T. Wu *et al.*, “Neodb: a comprehensive neoantigen database and discovery platform for cancer immunotherapy,” *Database*, vol. 2023, p. 41, Aug. 2023, doi: 10.1093/DATABASE/BAAD041.

[13] J. Xia *et al.*, “NEPdb: A Database of T-Cell Experimentally-Validated Neoantigens and Pan-Cancer Predicted Neoepitopes for Cancer Immunotherapy,” *Front Immunol*, vol. 12, 2021, doi: 10.3389/fimmu.2021.644637.

[14] K. Koyama, K. Hashimoto, C. Nagao, and K. Mizuguchi, “Attention network for predicting T cell receptor-peptide binding can associate attention with interpretable protein structural properties,” *bioRxiv*, p. 2023.02.16.528799, Jan. 2023, doi: 10.1101/2023.02.16.528799.
